# Damned If You Drain, Damned If You Don't: A Case of Pericardial Decompression Syndrome

**DOI:** 10.7759/cureus.9606

**Published:** 2020-08-07

**Authors:** Swathi Rao, Daniel Alcantar, Diana Espinoza, Robert Lichtenberg

**Affiliations:** 1 Internal Medicine, MacNeal Hospital, Berwyn, USA

**Keywords:** pericardiocentesis, pericardial decompression syndrome, cardiogenic shock, right heart failure, cardiac tamponade, pericardial window, pericardial effusion, pericardium, heart failure

## Abstract

Pericardial decompression syndrome (PDS) is an unusual clinical scenario with a reported incidence of 5% in all surgical or percutaneously managed pericardial tamponade patients. It is defined as a paradoxical hemodynamic instability leading to left ventricular (LV), right ventricular (RV), or biventricular dysfunction. An 84-year-old female with a history of a chronic pericardial effusion presented with symptoms of tamponade. She had had multiple prior admissions with an extensive and unyielding workup for the etiology of her pericardial effusion. During the present admission, a transthoracic echocardiogram (TTE) confirmed an augmenting pericardial effusion causing cardiac tamponade. She underwent a pericardial window with the removal of 1.2 liters of serous fluid. Postoperatively, she went into cardiogenic shock from right heart failure. Unfortunately, there also was re-accumulation of the pericardial effusion and worsening hemodynamic instability. Due to her poor prognosis, she was transitioned to comfort care. Although the etiology of PDS is unknown, it has been theorized to be caused by an imbalance of sympathetic-parasympathetic states after a rapid decompression. Currently, there are no clear guidelines or recommendations regarding the quantity of fluid that can be removed safely. More awareness leading to a more cautious and staged pericardial drainage might be the required solution.

## Introduction

Pericardial decompression syndrome (PDS) is an unusual clinical scenario with a reported incidence of 4.8% in all surgically or percutaneously managed cardiac tamponade patients [1]. It is defined as a paradoxical hemodynamic instability after drainage leading to left ventricular (LV), right ventricular (RV), or biventricular dysfunction. In this report, we present a case where the treatment turned out to be more of a curse than a cure.

## Case presentation

**Figure 1:**
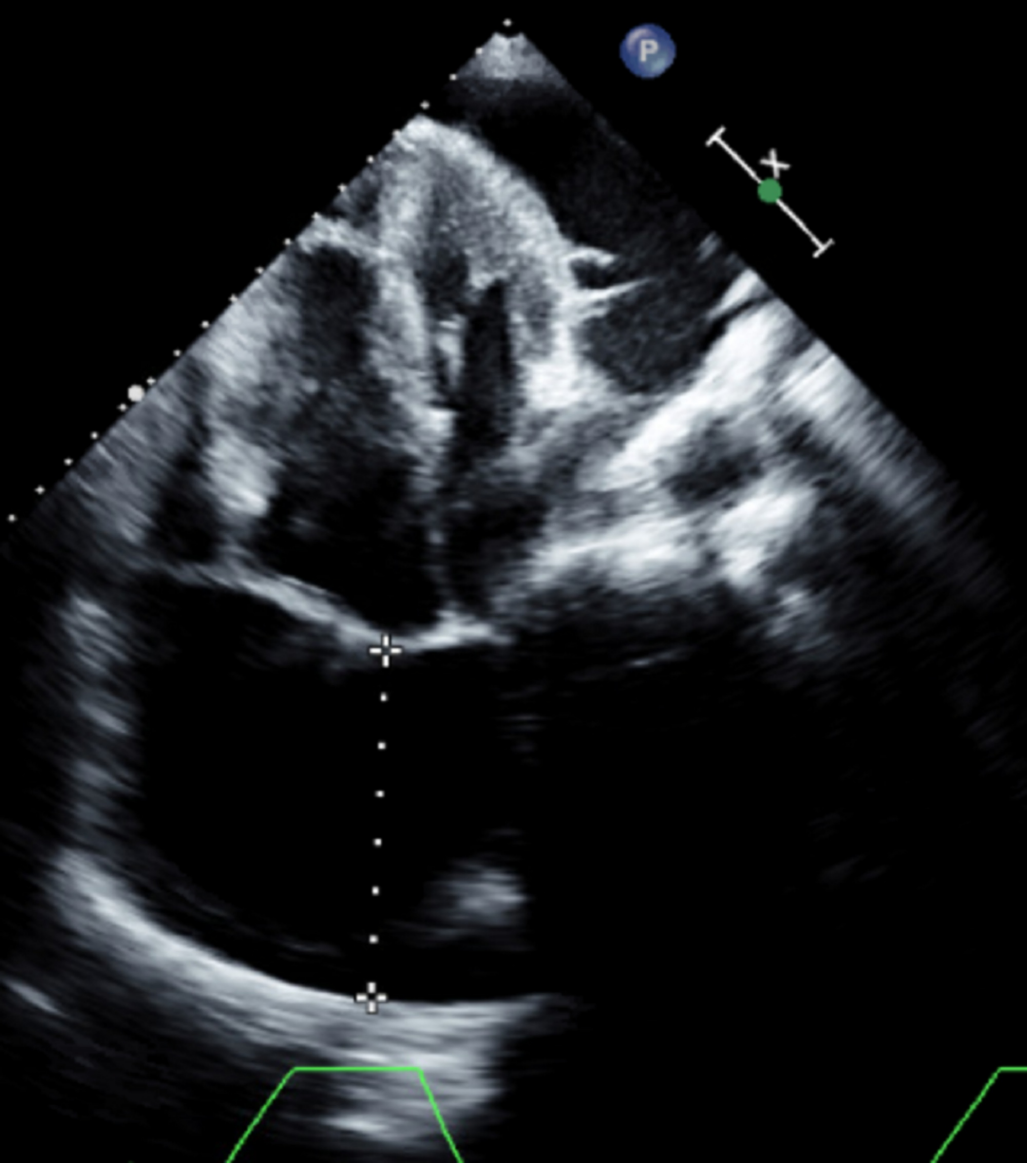
Apical four-chamber view by transthoracic echocardiogram at admission showing the large pericardial effusion

**Figure 2:**
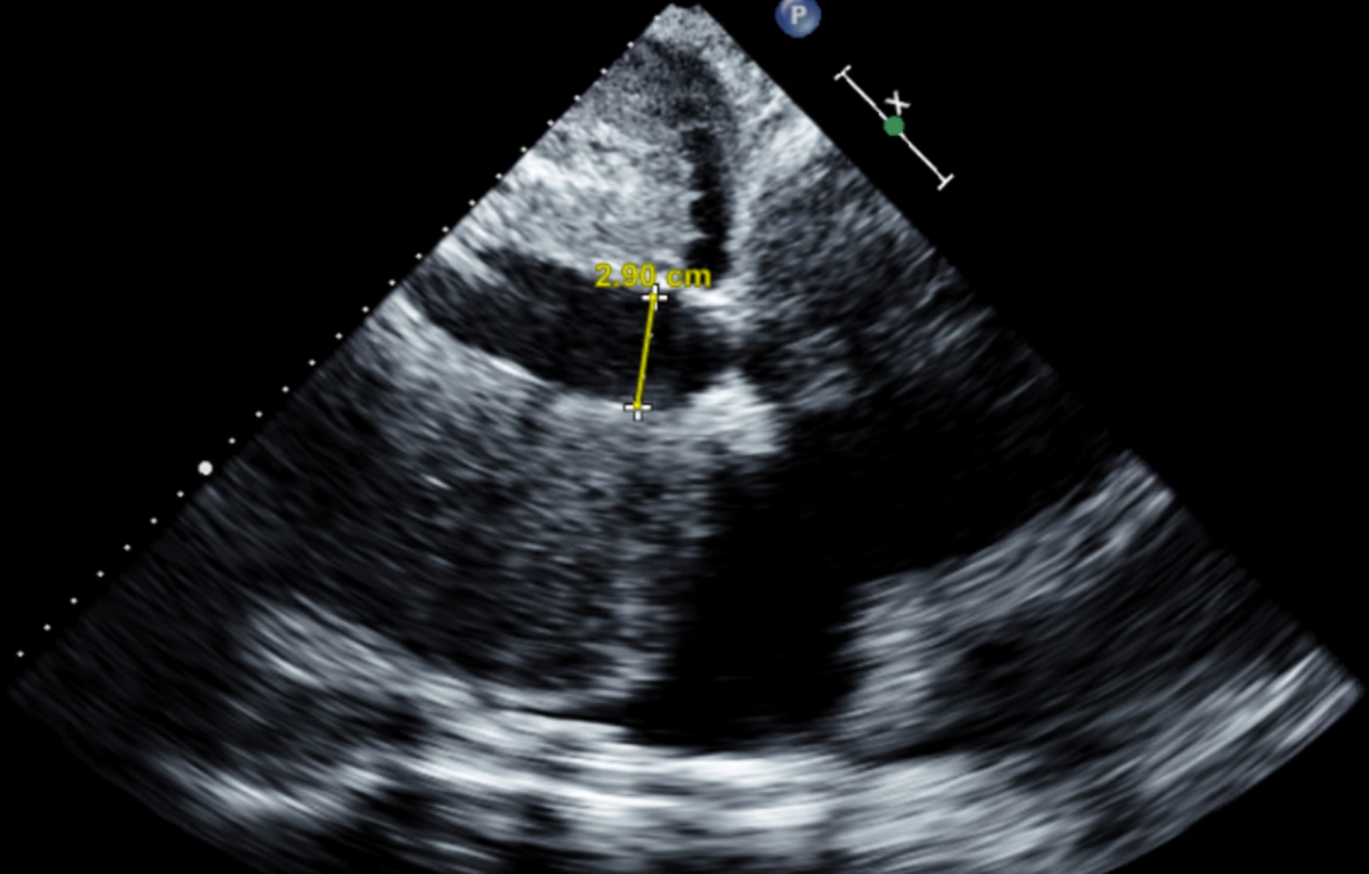
Dilated inferior vena cava at 29 mm as seen on transthoracic echocardiogram

**Figure 3:**
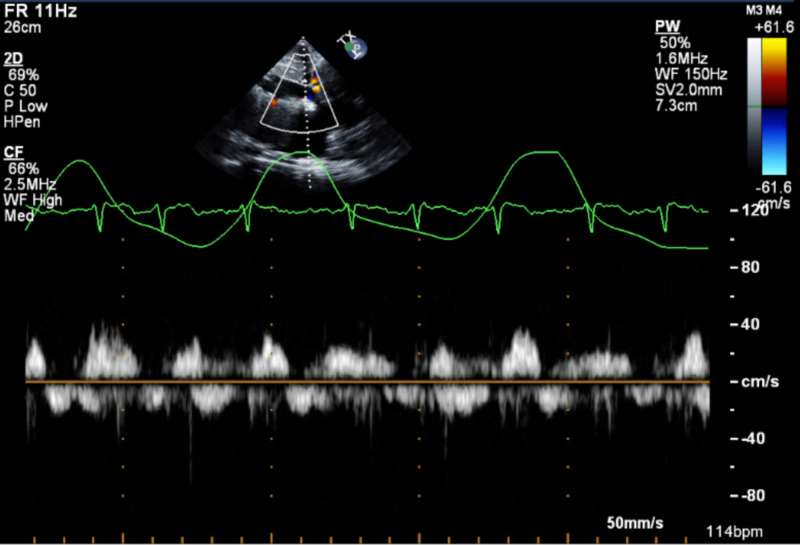
Pulsed wave Doppler echocardiogram at admission showing the blunted respirophasic motion of inferior vena cava

**Figure 4:**
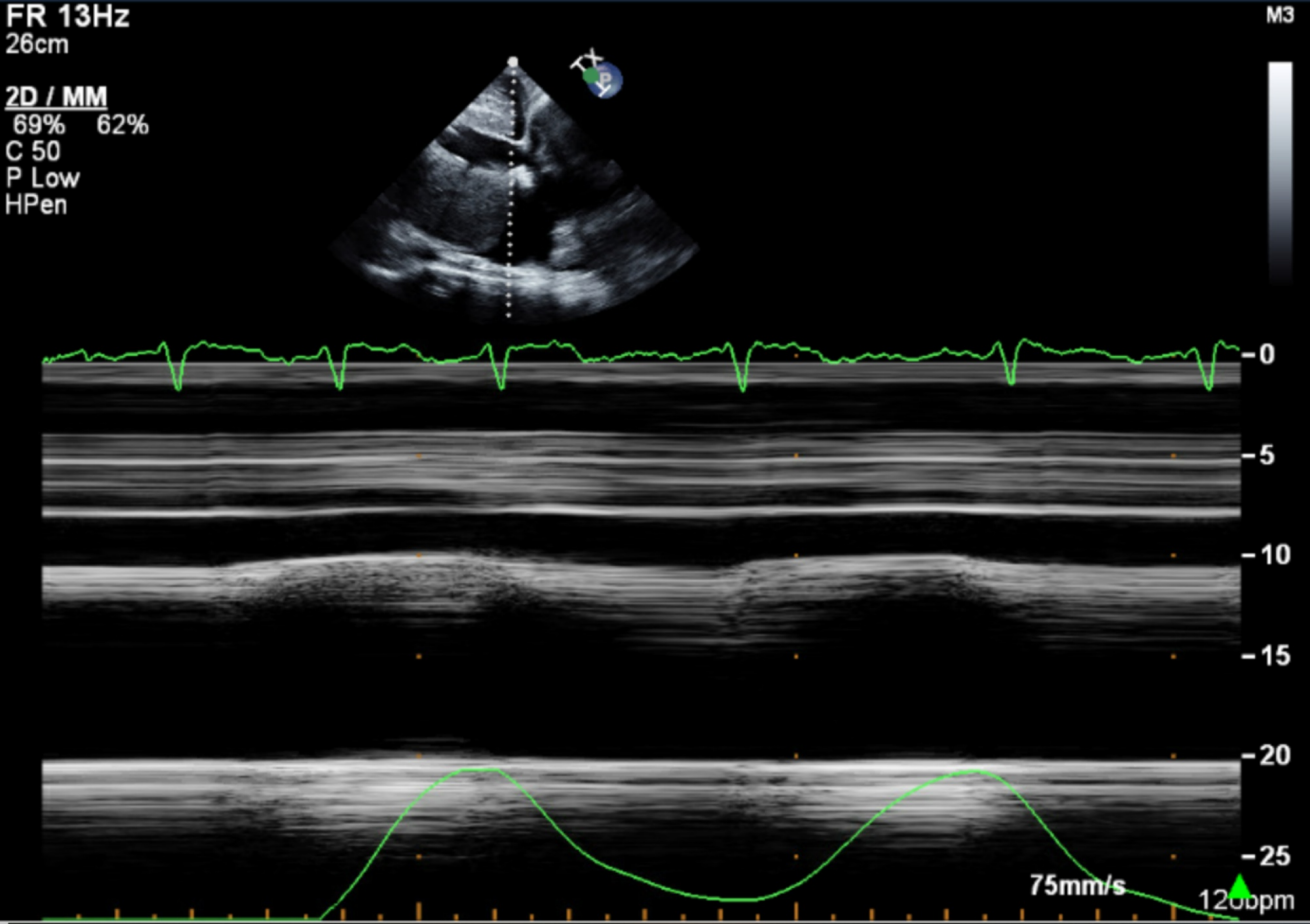
M-mode on transthoracic echocardiogram at admission showing non-compressibility and blunted respirophasic motion of inferior vena cava

An 84-year-old woman with a past medical history of hypertension, hyperlipidemia, heart failure with preserved ejection fraction (HFpEF), severe pulmonary hypertension (pulmonary arterial systolic pressure of 76 mmHg), and chronic pericardial effusion of unclear etiology presented to the ED with shortness of breath of sudden onset that had started at rest and had quickly become severe. She had a chronic large but hemodynamically stable pericardial effusion. An extensive workup for the etiology of her pericardial effusion had been stubbornly unrevealing. An elective pericardial window formation had previously been planned as she had been hemodynamically stable. During this admission, she was in moderate respiratory distress with sinus tachycardia to 105 beats/min, respiratory rate of 30 times/min, and oxygen saturation of 85% on room air. Physical examination revealed bilateral leg edema and jugular venous distension with muffled heart sounds. A bedside transthoracic echocardiogram (TTE) showed a new early diastolic RV collapse with a large pericardial effusion (Figure 1, Video 1). The inferior vena cava was dilated at 29 mm (Figure 2), was non-compressible, and had a blunted respirophasic motion (Video 2, Figures 3, 4). She underwent an emergent pericardial window with the removal of 1.2 liters of serous fluid and adequate clinical improvement. She was followed up closely in the intensive care unit (ICU) postoperatively. Fluid studies and an intraoperative pericardial biopsy yet again failed to reveal any pathology. On hospital day two, she became hypotensive to 85/40 mmHg with altered mental status. She subsequently went into cardiogenic shock complicated by pulmonary edema and hypoxic respiratory failure requiring intubation, diuretics, and inotropes. RV systolic failure and severe right atrial dilation were confirmed by a repeat TTE (Video 3). Over the next few days, there was worsening hemodynamic instability. Given her poor prognosis, the decision to transition her to comfort care was made by her family.

**Video 1:** Early right ventricular diastolic collapse and pericardial effusion at admission on apical four-chamber view of transthoracic echocardiogram

**Video 2:** Doppler echocardiogram of inferior vena cava at the time of admission IVC: inferior vena cava

**Video 3:** Apical four-chamber view on transthoracic echocardiogram at the time of cardiogenic shock showing severe right atrial dilation and right ventricular systolic dysfunction RV: right ventricular

## Discussion

PDS should be taken into account when considering treatment for patients with large pericardial effusions. Although the etiology of PDS is unknown, it has been known to cause a variety of complications including LV failure, RV failure, or both [1]. In our case, we highlight the occurrence of PDS with RV failure after the removal of a large, chronic pericardial effusion of unknown etiology.

The heart is surrounded by the pericardium composed of two thin layers, a serous visceral and a fibrous parietal layer, typically containing 50 mL of serous fluid. Pericardial effusion occurs when the volume of fluid exceeds this normal amount [2]. The causes of pericardial effusions are numerous and can be divided into two categories: inflammatory and non-inflammatory. Common inflammatory etiologies include infectious, autoimmune, uremic, or drug hypersensitivity. Non-inflammatory etiologies include neoplastic, metabolic, traumatic, iatrogenic causes, or post-radiation [2].

In a recent systemic review by Klimis et al., which reported all cases of PDS, two-thirds of patients (24/35) presented with cardiogenic shock [3]. Multiple variations of cardiogenic shock were noted: 40% with LV failure, 9% with RV failure, and 20% with biventricular failure. The causes of the effusion were unknown in the majority of cases (42%) with the second most common cause being a malignant effusion (40%). The volume of pericardial fluid drained ranged from 450 to 2,100 mL (mean: 888 mL). Interestingly, 29% of patients died and the mortality risk was found to be higher in patients who underwent surgical pericardiotomy [3]. In those who survived with LV impairment, TTE follow-up showed a return of normal LV function [4]. Similarly, reports of RV dysfunction in those who survived also showed a return of normalized RV function.

Currently, the mechanism of PDS remains unclear. However, there are multiple theories reported as to why PDS would occur. Vandyke et al. suggest that LV dysfunction following pericardiocentesis may be related to the interventricular volume mismatch in the setting of elevated systemic vascular resistance and tachycardia [5,6]. However, Braverman et al. suggest that ventricular failure is caused by myocardial stunning medicated by a decrease of epicardial coronary blood flow due to an increase in pericardial pressure resulting from the effusion [5,7]. Other possible causes include alleviation of a high catecholamine state and unmasking of LV dysfunction, as well as sudden vasodilatory hemodynamic collapse due to the sympathetic-parasympathetic system imbalance [5]. The sudden increase in RV preload following decompression also can overwhelm the RV, resulting in ventricular dilation and RV failure [5,6], which may have been the mechanism in our case. 

Pericardiocentesis can be a life-saving measure in the treatment of cardiac tamponade. As per current guidelines, an absolute indication for emergent pericardiocentesis is a hemodynamically compromised patient in cardiac tamponade [8]. Over the years, the procedural techniques with echo, CT, or fluoroscopy-guided methods have been perfected to try and minimize complications. Rates of major complications in observational studies have ranged from 0.3 to 3.9%, while that of minor complications have ranged from 0.4 to 20% [9]. Major complications include cardiac chamber or coronary vessel puncture (which may present as an initially silent hemopericardium), puncture of abdominal viscera, pneumopericardium, pneumothorax, ventricular arrhythmias, and, rarely, PDS. Minor complications include transient vasovagal bradycardia and hypotension, supraventricular arrhythmias, mild pneumothorax, and pleuropericardial fistulas.

The predictors for the occurrence of PDS are vague. In an observational retrospective study, the mortality rate from PDS was seen to be as high as 29%. One of the methods routinely used to prevent the incidence is to perform a staged pericardiocentesis, initially draining only enough fluid to reverse tamponade followed by drainage of the remaining fluid slowly over the next few hours [9].

## Conclusions

Pericardiocentesis has been the life-saving measure to turn to during cardiac tamponade. But sudden decompressions of large pericardial effusions might also lead to harm from PDS. Although the true incidence of PDS is unknown, clinicians should be aware of it when discussing surgical or percutaneous treatment for pericardial effusions. More awareness might lead to a more cautious approach and staged pericardial fluid drainage. This could very well mean the difference between life and death for patients.
